# Posttraumatic Skin and Soft-Tissue Infection due to* Pseudomonas fulva*

**DOI:** 10.1155/2016/8716068

**Published:** 2016-09-26

**Authors:** Fernando Cobo, Gemma Jiménez, Javier Rodríguez-Granger, Antonio Sampedro

**Affiliations:** Department of Microbiology, Complejo Hospitalario Universitario de Granada, Granada, Spain

## Abstract

We report a case of posttraumatic skin and soft-tissue infection in a patient with a left thigh wound after a traffic accident.* Pseudomonas fulva *was isolated from a wound aspirate and was identified to the species level by Maldi-tof. The patient responded to drainage, debridement of wound, and two weeks of intravenous antibiotic therapy. Follow-up after 3 weeks was satisfactory with healthy cover of the injured area.

## 1. Introduction

*Pseudomonas fulva *was first described in 1963 and belongs to fluorescent group of* Pseudomonas* [1]. This microorganism is mainly recovered from rice seed samples, rice, and petroleum fields and oil brine and from the gills of some mollusks [2]. These bacilli are an uncommon pseudomonal opportunistic pathogen causing very rare infection in human population. Until now, only two infections have been documented in humans: an isolate causing meningitis in a 2-year-old girl after placement of a cerebral shunt system [3] and an isolate causing bacteremia in a 56-year-old man who suffered a trauma [4]. In our knowledge, we are describing the third case of infection by* P. fulva* in a patient with a trauma due to a traffic accident.

## 2. Case Report

A 73-year-old man presented to the emergency department of our hospital due to a right hip fracture because of a traffic accident. The patient had also a lacerated wound on the left thigh with underlying bone injury at this level. After initial assessment and management, a stabilizing surgery followed by cleaning and suturing of the wound was performed. After 48 h of admission, a serohematic exudate together with swelling and erythematous changes was observed in the wound. A new cleaning and debridement was carried out, taking at this moment an aspirate from the exudate for microbiologic study. Empirical treatment with teicoplanin (6 mg/kg/i.v daily) plus cefepime (1 gr/i.v/12 h) was then started.

After centrifugation, the sample was inoculated in aerobic and anaerobic blood agar (BD Columbia Agar 5% Sheepblood®, Becton Dickinson), chocolate agar (BD Choco Agar, Becton Dickinson), and thioglycollate broth (BD*™* Fluid Thioglycollate Medium), all incubated at 37°C. Approximately, 1 mL of the sample was inoculated into an aerobic blood culture bottle (BACTEC, 9240 BD, Becton Dickinson, Franklin Lakes, NJ, USA). A Gram stain of the sample revealed no microorganisms. After 18 hours of incubation, both thioglycollate broth and blood culture bottle were positives, and subcultures in the same plates were carried out. A Gram stain of both fluids revealed Gram-negative bacilli. After 18 hours of incubation, pathogen growth was observed on all plates. The strain was small, straightedged, and slightly yellowish. The oxidase was clearly positive. The Gram-negative rod was identified by mass spectrometry (Bruker Biotyper, Billerica, MA, USA) as* P. fulva* (score 2.295), and susceptibility to this strain was then tested by a microdilution method (MicroScan, Beckman-Coulter Inc.). According the CLSI recommendations [5], the isolate was susceptible to aminoglycosides, ciprofloxacin, piperacillin-tazobactam, imipenem, and ceftazidime, whereas it was intermediate to meropenem and cefepime. The antibiotic therapy was changed to intravenous ceftazidime (2 gr/8 h) for 2 weeks, and follow-up after 3 weeks was satisfactory with healthy cover of the injured area.

## 3. Discussion

*P. fulva* is a Gram-negative rod which belongs to genus* Pseudomonas *and shares genotypic characteristics with the fluorescent* Pseudomonas* group, above all with* P. putida. *This species was first described in 1963 [1], but recently seven of these strains were recharacterized [6]. These bacilli produce a yellow but no fluorescent pigment and also produce arginine hydrolysis and catalase [6]. Oxidase activity is variable, being positive in three cases described by Uchino et al. [6], in one case described by Seok et al. [4], and in our case. In only an isolate described by Almuzara et al., oxidase test was negative [3]. The variable oxidase reaction may be a phenotypic characteristic of this species and, like in* Aeromonas*, may be negative if performed on strains from differential media such as MacConkey agar but positive if performed on strains from nondifferential media [7]. In blood agar, these strains are small, round, and yellowish.

*P. fulva *is mainly isolated from environmental sources, above all rice paddies, and rarely affects humans. Until now, only three cases of* P. fulva* have been reported in the medical literature. One case was isolated from sputum in a patient with cystic fibrosis [8], but the clinical significance of this report was not definitively clarifying. However, the remaining two cases had clinical significance. In 2010, Almuzara et al. [3] reported the first case of human infection due to* P. fulva* in a 2-year-old female with acute meningitis after placement of shunt system. The isolate shows a VIM-2 carbapanemase production and the patient was treated with piperacillin-tazobactam, colistin, and ciprofloxacin. At the same year, Seok et al. [4] reported a case of bacteremia due to this bacterium in a patient hospitalized due to a trauma. This patient was treated with piperacillin-tazobactam and clindamycin with satisfactory outcome. Due to its rarity, treatment of choice to* P. fulva* has not been established, so susceptibility testing to the isolates should be performed in order to give the correct therapy. Moreover, care should be taken because this species may acquire resistance genes such as carbapenemases [3].

Little is known about the transmission of this infection to humans, but the existence of traumatisms and/or placement of medical devices may help to develop the infection from the environmental sources.

In summary,* P. fulva* is a very rare infection in humans and only three cases have been described until now. Although this bacterium is usually an environmental microorganism, physicians and microbiologists should be aware of this infection above all in patients with posttraumatic infections.
